# A non-linear ensemble model-based surgical risk calculator for mixed data from multiple surgical fields

**DOI:** 10.1186/s12911-021-01450-9

**Published:** 2021-07-30

**Authors:** Ruoyu Liu, Xin Lai, Jiayin Wang, Xuanping Zhang, Xiaoyan Zhu, Paul B. S. Lai, Ci-ren Guo

**Affiliations:** 1 School of Computer Science and Technology, Xi’an Jiaotong University, Xi’an, 710049 China; 2 Department of Surgery, The Chinese University of Hong Kong, Hong Kong, China; 3 Department of Tumor Gynecology, Fujian Medical University Cancer Hospital and Fujian Cancer Hospital, Fuzhou, 350014 China

**Keywords:** Surgical risk calculator, Gradient boosting decision tree, Machine learning, Clinical decision support system

## Abstract

**Background:**

The misestimation of surgical risk is a serious threat to the lives of patients when implementing surgical risk calculator. Improving the accuracy of postoperative risk prediction has received much attention and many methods have been proposed to cope with this problem in the past decades. However, those linear approaches are inable to capture the non-linear interactions between risk factors, which have been proved to play an important role in the complex physiology of the human body, and thus may attenuate the performance of surgical risk calculators.

**Methods:**

In this paper, we presented a new surgical risk calculator based on a non-linear ensemble algorithm named Gradient Boosting Decision Tree (GBDT) model, and explored the corresponding pipeline to support it. In order to improve the practicability of our approach, we designed three different modes to deal with different data situations. Meanwhile, considering that one of the obstacles to clinical acceptance of surgical risk calculators was that the model was too complex to be used in practice, we reduced the number of input risk factors according to the importance of them in GBDT. In addition, we also built some baseline models and similar models to compare with our approach.

**Results:**

The data we used was three-year clinical data from Surgical Outcome Monitoring and Improvement Program (SOMIP) launched by the Hospital Authority of Hong Kong. In all experiments our approach shows excellent performance, among which the best result of area under curve (AUC), Hosmer–Lemeshow test (${{\mathrm{HL}}}_{\hat{c}}$) and brier score (BS) can reach 0.902, 7.398 and 0.047 respectively. After feature reduction, the best result of AUC, ${\mathrm{HL}}_{\hat{c}}$ and BS of our approach can still be maintained at 0.894, 7.638 and 0.060, respectively. In addition, we also performed multiple groups of comparative experiments. The results show that our approach has a stable advantage in each evaluation indicator.

**Conclusions:**

The experimental results demonstrate that NL-SRC can not only improve the accuracy of predicting the surgical risk of patients, but also effectively capture important risk factors and their interactions. Meanwhile, it also has excellent performance on the mixed data from multiple surgical fields.

## Background

In 2008, the United States Department of Health and Human Services (HSS) reviewed the medical records of hospital inpatients, and pointed out in the investigation report that 180,000 people died from medical errors in medical insurance patients alone [1]. In 2013, deaths caused by medical errors have become the third leading cause of death in the United States [2]. Among them, surgery, as one of the most important treatment methods in modern healthcare, accounts for a large proportion of deaths due to medical errors caused by misestimation of surgical risks. According to statistics, more than 234 million operations are performed every year around the world, but unfortunately, as many as 3% of these operations will bring bad results and cause 1 million deaths [3]. What is even more regrettable is that nearly half of them can be avoided or prevented [4]. Therefore, how to improve the accuracy of surgical risk prediction and reduce the number of surgical errors has become one of the urgent tasks of the current medical domain.

Surgical risk prediction is an important part of the clinical decision support system [5], which is of great significance for controlling surgical medical errors and ensuring the life safety of patients. An accurate surgical risk prediction system can not only help surgeons provide patients with better surgical options [6], better perioperative management [7], and potential opportunities to improve outcomes [8], but also help to minimize medical costs [7] and allocate medical resources reasonably [9].

However, establishing an accurate system to predict the risk of postoperative death remains an ongoing challenge [10]. Surgical risk calculator, as an important tool for doctors and patients to make joint decision on treatment options [11], is a core idea to deal with it [12]. An ideal surgical risk calculator should be one that is simple, objective, reproducible, accurate [9], and learns knowledge from patient data by using a series of empirical risk models to provide patients with surgical risk prediction results in a specific time period [13]. The results not only strengthen the communication between doctors and patients to help patients’ informed consent [14], but also provide doctors with better decision-making suggestions with patients’ specific information [15].

The research on methods of predicting and evaluating surgical risks has been going on since the 1960s. In 1963, the American Society of Anaesthesiologists (ASA) grade was proposed [16] and widely used and developed, which is simple to use but too subjective [10]. Goldman et al. [17] used 9 preoperative variables to develop a cardiac risk index in 1977, and was revised by Lee et al. [18] in 1999 to reduce the number of variables to 6. In 1981, the Acute Physiology and Chronic Health Evaluation (APACHE) scoring system [19] was proposed to assess the severity of the disease and predict mortality by using data obtained within 24 hours after admission to the ICU. After that, APACHE was updated and revised three times [20–22], which improves the accuracy of prediction but increase the burden of data collection [23], making it too complex to be considered suitable for general surgery [10]. The Charlson index [24] proposed in 1987 can predict the morbidity and mortality of patients in multiple different surgical cohorts by using preoperative factors, but it lacks subjectivity in the evaluation of patient comorbidities [9]. POSSUM (Physiological and Operative Severity Score for the enUmeration of Mortality and morbidity), a physiological and surgical severity scoring system proposed by Copeland et al. [25] in 1991, integrated preoperative, intraoperative, and postoperative factors (12 physiological indicators and 6 surgical measures) to improve the completeness of predictions. Prytherch et al. believed that POSSUM has too high a prediction of the probability of low-risk patients dying within 30 days after surgery. Instead, they used the same variables to conduct linear analysis of the original POSSUM equation and proposed P-POSSUM (Portsmouth-POSSUM) [26], which has been shown to have a more accurate mortality prediction [27]. However, some studies reported that the accuracy of P-POSSUM fluctuates greatly [28]. In 2003, Prytherch et al. [29] proposed the Biochemistry and Haematology Outcome Model (BHOM), using fewer objective variables to predict postoperative mortality. In 2008, the Hospital Authority of Hong Kong launched a Surgical Outcome Monitoring and Improvement Program (SOMIP), which estimates the survival risk of patients through statistical adjustments to different preoperative factors [30], to annually audit the surgical performance of all public hospitals in the territory [31].

In 2013, the American College of Surgeons National Surgical Quality Improvement Program (ACS-NSQIP) developed the first universal risk calculator in the United States [32] for improving the quality of surgical procedures and predicting surgical risk. NSQIP collected high-quality, standardized data from more than 1.4 million operations performed in about 500 hospitals in the US [33], and then used logistic regression model to quantify and predict the surgical risk of patients within 30 days [9]. Meanwhile, the database it used is constantly updated [15]. Although NSQIP is supported by a large and extensive database, its effectiveness in other surgical specialties has not been definitively verified, because it is derived from a specific risk calculator designed for patients undergoing colorectal surgery [32]. The risk calculators develope on this data set also included the Surgical Risk Preoperative Assessment System (SUPPAS) [34], which provided 8 aspects of preoperative risk prediction of adverse postoperative outcomes within 30 days. ACS-NSQIP exerted great influence since it was proposed and has been applied in many research fields [12, 35, 36]. As the research continues, more and more disease-specific risk assessment models or systems were developed and made great contribution, such as in the study of aneurysmal subarachnoid haemorrhage [37], radical cystectomy [38] and prostate biopsy [39]. At the same time, many portable simple risk calculators based on mobile devices have been developed [40] as well.

The risk calculators above are based on linear models with logistic regression as the core, and some researchers have tried to add non-linear models in them. In order to effectively explain the non-linear correlation between physiological factors and results, Johnson et al. [41] used a heuristic algorithm to select the minimal set of variables, called the Oxford Acute Severity of Illness Score (OASIS), to assess the severity of the patient’s condition. Savin et al. [42] identified the risk factors of Healthcare-Associated Ventriculitis and Meningitis (HAVM) by taking the intersection of the variables screened by 5 linear and non-linear methods. Unfortunately, these two works only use non-linear methods in feature selection, but not in risk prediction.

Although all the models or systems mentioned above make outstanding contribution, they have one common limitation: the risk prediction model is linear. Despite the traditional methods based on linear models are simple in form, intuitive in the correlation between/among factors and easy to be explained by non-experts [41], there are also many disadvantages. Firstly, it requires variables to have some degree of correlations, and often require subjective modeling decisions (e.g., which interaction items to include) [41]. Secondly, its sensitivity to data noise and multicollinearity may lead to misleading conclusions [43]. Thirdly, the learning ability of linear models is limited [44], so that most of the existing methods have already encountered the performance bottleneck and difficult to further improve the prediction accuracy [45]. Finally and most importantly, it is hardly for linear models to learn the high-dimensional non-linear correlations between/among risk factors, which play more important roles in the complex physiological process of the human body [46]. At present, many studies have proved that the interactions between/among factors can importantly affect the occurrence, development and prognosis of complex diseases [47–51], which is likely to be in a non-linear way rather than simply adding up [41]. Even though some linear methods can deal with non-linear interactions by adding high-dimensional cross product terms [52–54], the specification of the order of cross product terms and the relevant interpretation may be the practical difficulty for building the surgical risk calculator [55, 56]. Therefore, predicting surgical risk based on a linear model does not conform to the fact that non-linear interactions dominate in the actual physiological process, and it will lead to more serious performance bottleneck.

In this paper, we presented a non-linear surgical risk calculator (NL-SRC) for mixed data from multiple surgical fields, in which the Gradient Boosting Decision Tree (GBDT) model is used to obtain accurate postoperative risk prediction by capturing the non-linear interactions between/among risk factors. To the best of our knowledge, this study is the first one to apply it as a core prediction model for surgical risk. Therefore, the first innovation of this paper is to try to use a non-linear ensemble model to break through the performance bottleneck of existing methods. However, it is difficult to achieve good results by simply using GBDT to predict the surgical risk on real surgical clinical data, because some characteristics of the data will make GBDT under-fit and learn wrong knowledge, and thus lead to wrong conclusions. These characteristics mainly include: (1) mixed data types; (2) uneven quality of data from different hospitals and duplicate records and (3) missing values. Therefore, the second innovation of this paper is to design an applicable pipeline to cope with such challenges when constructing the risk calculator. We made many attempts and exploration on data preprocessing and missing value filling methods. Specifically, for characteristic 1 and 2, we tried a variety of different risk factor combination strategies and data encoding methods, and finally determined a data preprocessing process suitable for GBDT that gives priority to categorical features and performs global encoding; for characteristic 3, we tried many missing value filling methods, and finally chose the filling method similar to the missing value processing in XGBoost. In addition, considering that a surgical risk calculator must be practice-oriented, we also try to enhance the practicality of our approach by two ways. One is that we designed three different training modes to ensure users can flexibly choose the way to use our approach according to their own sample size, and the other one is that we screened out the important risk factors based on the feature importance of tree-model to reduce the complexity of GBDT and improve the practicability of our approach. In order to prove the superiority of our approach compared with baseline models and similar models, we also built multiple traditional models (logit, Support Vector Machines, Classification and Regression Tree) and similar ensemble models (Random Forest, XGBoost) for comparative experiments, among which the logit model is the most widely used baseline model in previous studies for surgical risk prediction [32, 41, 57–60]. Experiments conducted on the three years (2010–2012) of real clinical data of SOMIP show that NL-SRC performs well in each training mode and outperforms all other models. In addition, considering that one of the obstacles to clinical acceptance of surgical risk calculators is that models are too complex to be used in practice [61], we reduced the number of input risk factors according to the importance of them in GBDT. Subsequent experiments prove that our approach still obtains good results even after removing most of the risk factors. The results demonstrate that our approach has advantages in all aspects. Finally, we also analyzed the selected risk factors and the interactions between/among them, whose results prove that our approach is reasonable.

## Methods

Ensemble learning is a kind of algorithm that builds multiple base learners and combines the outcomes of them to perform learning tasks, which can be divided into the “bagging” algorithms (such as Random Forest) and the “boosting” algorithms (such as GBDT [62]). The boosting algorithm combines weak learners by performing multiple iterations on the same data set to jointly build a strong learner [63]. As the representative algorithm of them, GBDT takes the regression tree model of Classification and Regression Tree (CART) [64] as the base learner, uses negative gradient as the approximate method of the steepest descent algorithm to optimize the loss function and fit the base learner, and finally integrates the trees with Gradient Boosting Machine (GBM) to build the model. Some works have pointed out that tree-based machine learning algorithms are effective methods to study surgical risk factors [42], and boosting-based methods have been applied in clinical medical research and have achieved excellent results [42, 65].

In this part, we will first brief the regression tree of CART, and then introduce the GBDT algorithm based on the restatement of the problem of surgical risk prediction. After that, how to calculate the feature importance in tree models will be explained, and finally the main parts of the pipeline of our approach will be introduced.

### The regression tree of CART

Decision tree is a classic and widely used machine learning model, which represents a mapping between object properties and object values. In general, a decision tree contains a root node, several internal nodes, and several leaf nodes. The root node contains the entire samples. Each leaf node represents a decision result that contains samples of the same category. With the exception of leaf nodes, the samples contained in each other node will be divided into their respective child nodes according to the corresponding partition rules, thereby forming a complete decision-making judgment path from root node to leaf nodes. The key of decision tree is how to choose the optimal split attribute. As the partition process progresses, we want the “purity” of the samples contained in branch nodes keep increasing, that is, to be in the same category as possible.

CART is a classic decision tree algorithm proposed by Breiman et.al [64] in 1984, two models, classification tree and regression tree, were designed for classification problems and regression problems, respectively. Since the base learner used by GBDT is the regression tree, we only introduced the basic principle of it here. Regression tree usually uses least squares deviation (LSD) or least absolute deviation (LAD) as the loss function. Here, we take LSD as an example to brief.

For a given data set {$\varvec{x}_i$, $y_i$}$^N_1$, where $\varvec{x}_i$ is the *i*-th input data and $y_i$ is the corresponding label, that is, whether the patient died within 30 days after the surgury (death is 1, otherwise 0). As a recursive binary tree algorithm, regression tree partitions the data space into multiple subspaces (hereinafter referred to as “units”). Therefore, we assume that an initial regression tree model $h(\varvec{x})$ partitions the data space into *M* units, denoted as {$\varvec{R}_m$}$^N_1$, the loss function on the *m*-th unit can be written as:

1 $$\begin{aligned} L_m=\sum _{\varvec{x}_i\in \varvec{R}_m}(y_i-(h(\varvec{x}_i))^2 . \end{aligned}$$

We hope to find an optimal mapping function $h^*(\varvec{x})$ that minimizes Eq. 1. It is easy to know that when $h(\varvec{x}_i\mid \varvec{x}_i\in \varvec{R}_m)$ is equal to the mean of the actual values of all samples in $\varvec{R}_m$ can achieve the target [64], that is, $h(\varvec{x}_i\mid \varvec{x}_i\in \varvec{R}_m)=C_m=\mathrm{AVE}(y_i\mid \varvec{x}_i\in \varvec{R}_m)$. Then Eq. 1 can be rewritten as $L_m=\sum _{\varvec{x}_i\in \varvec{R}_m}(y_i-C_m)^2$.

After determining the basic form of the loss function, we can choose the optimal partition strategy for regression tree with the goal of minimizing it. Assuming that we take the risk factor *v* as the split attribute and a certain value *s* of it as the split point to split the original data set, two units and the mean values of them can be obtained:

$$\begin{aligned} \varvec{R}_1(v,s)=\{\varvec{x}\mid \varvec{x}^v\le s\},\qquad \varvec{R}_2(v,s)=\{\varvec{x}\mid \varvec{x}^v>s\},\\ C_1=\mathrm{AVE}(y_i\mid \varvec{x}_i\in \varvec{R}_1(v,s)),\qquad C_2=\mathrm{AVE}(y_i\mid \varvec{x}_i\in \varvec{R}_2(v,s)). \end{aligned}$$

Therefore, we traverse all risk factors and all the values of each risk factor to achieve the optimal partition of the sample set at this depth by finding out the combination (*v*, *s*) that minimizes the loss function, namely:

2 $$\begin{aligned} \min \limits _{v,s}\left[ \min \limits _{C_1}\sum _{\varvec{x}_i\in \varvec{R}_1(v,s)}(y_i-C_1)^2 + \min \limits _{C_2}\sum _{\varvec{x}_i\in \varvec{R}_2(v,s)}(y_i-C_2)^2\right] . \end{aligned}$$

Suppose the optimal split combination obtained according to Eq. 2 is $(v^*,s^*)$, the corresponding unit output value can be calculated as $C_m^*=\frac{1}{N_m}\sum _{\varvec{x}_i\in \varvec{R}_m(v^*,s^*)}y_i,m=1,2$. The optimal regression tree mapping function can be obtained by repeating the above process on the subunits until the stop condition is satisfied, which is $h^*(\varvec{x})=\sum _{m=1}^{M}C_m^*\cdot I(\varvec{x}\in \varvec{R}_m)$.

Although decision tree algorithm has some advantages, its application range and effect are limited by its strong subjectivity and difficulty in solving the problems with large data volume or high complexity. Therefore, the ensemble algorithms based on decision tree was proposed and widely used. Ensemble learning algorithms are useful tools for performing multiple prediction tasks and can provide greater accuracy than traditional single machine learning models consistently [66].

### Gradient boosting decision tree algorithm

GBDT builds a new CART regression tree in each round of iteration and uses the negative gradient of the loss function to approximate the residual of the results in last iteration, and then fits the new tree built in this iteration by minimizing its loss function [44]. With the increase of the number of iterations, the residual generated in the training process will continuously decrease, the result thereby continuously approaching the true value.

#### Problem restatement based on gradient boosting

The purpose of machine learning is to maximize the reconstruction of unknown mapping relationships from data to results. To explain how GBM works clearly, we will start with a simple example [67]. Given a set of independent data $\varvec{X}$, assuming that $H_0$ is a model based on a decision tree that needs to be improved, and $\varvec{Y}$ is the corresponding label, it is easy to get $\varvec{Y} = H_0(\varvec{X})+error0$. We further fit a new decision tree model $H_1$ to predict *error*0 by $error0=H_1(\varvec{X})+error1$. Similarly, in each of the next steps, we predict the error of the last step in the same way, namely $error2=H_2(\varvec{X})+error1,\cdots \cdots$. When the stop condition is satisfied, we combine all the obtained models:

3 $$\begin{aligned} \varvec{Y}=errorI+\sum _{i=1}^{I}H_i(\varvec{X}). \end{aligned}$$

Generally, the performance of Eq. 3 will be better than the initial $H_0$, because the residual of each step is paid attention to and fitted.

The above example is a simple explanation of the basic idea of GBM, and then we will give a specific introduction in theory. Using the same symbolic representation as before and suppose the optimal mapping function is $f^*(\varvec{x})$. Given the loss function $\Psi (y, f(\varvec{x}))$, our target can be expressed as:

4 $$\begin{aligned} f^*(\varvec{x})=\arg \min \limits _{f(\varvec{x})}\Psi (y, f(\varvec{x})). \end{aligned}$$

Equation 4 can be rewritten into the form of expectation estimation [63]:

5 $$\begin{aligned} f^*(\varvec{x})=\arg \min \limits _{f(\varvec{x})}E_{\varvec{x}}(E_y(\Psi (y, f(\varvec{x})))\mid \varvec{x}). \end{aligned}$$

To make Eq. 5 tractable, we can restrict the search space of the mapping function to search optimal parameters [66] by $f^*(\varvec{x})=f^*(\varvec{x}, \varvec{\theta ^*})$. Rewrite Eq. 5 :

6 $$\begin{aligned} \varvec{\theta ^*}=\arg \min \limits _{f(\varvec{x})}E_{\varvec{x}}(E_y(\Psi (y, f(\varvec{x},\varvec{\theta })))\mid \varvec{x}). \end{aligned}$$

Adopt the addition model to combine base learners, given *T* iteration steps, the estimation of parameters can be written as $\varvec{\theta ^*}=\sum _{j=1}^{T}\varvec{\theta }_j^*$. The loss function on the given data set can be written as:

7 $$\begin{aligned} L(y,\varvec{\theta ^*}) = \sum _{i=1}^{N}\Psi (y_i, f(\varvec{x}_i,\varvec{\theta ^*})). \end{aligned}$$

Assume that the function of the newly built base learner in each iteration can be expressed as $h(\varvec{x},\varvec{\theta })$ in the parametric form, according to the additive ensemble principle, the collapsed result of the previous *t* iterations can be expressed as:

8 $$\begin{aligned} f_t(\varvec{x},\varvec{\theta }_t)=\sum _{j=1}^{t}\sum _{i=1}^{N}w_jh(\varvec{x}_i,\varvec{\theta }_j). \end{aligned}$$

where $w_j$ and $\varvec{\theta }_j$ are the weight and parameters of the base learner in the *j*-th iteration, respectively. According to the forward distribution algorithm of a tree, Eq. 8 can be rewritten as $f_t(\varvec{x},\varvec{\theta }_t)=f_{t-1}(\varvec{x},\varvec{\theta }_{t-1})+w_t h(\varvec{x},\varvec{\theta }_t)$, thus minimizing the loss function can be equivalent to:

9 $$\begin{aligned} (w_t,\varvec{\theta }_t)=\arg \min \limits _{w,\varvec{\theta }}\sum _{i=1}^{N}\Psi (y_i, f_{t-1}(\varvec{x}_i,\varvec{\theta }_{t-1})) +wh(\varvec{x}_i,\varvec{\theta }). \end{aligned}$$

When the loss function is a function that is easy to obtain residual, the gradient descent algorithm can be used for fast and simple optimization. In practice, however, some specific or custom loss functions are difficult to make it. At this time, the negative gradient of loss function for each iteration can be regarded as an approximation of the residual of last iteration, and take minimizing it as the optimization target for the current iteration [66]. Then, integrate the outcomes of the base learners built before to achieve the effect of gradient boosting [63].

Using the same symbolic representation as before, according to Eqs. 6 and 7, the gradient of the loss function in the *t*-th iteration is:

10 $$\begin{aligned} G_t(\varvec{x})=E_y\left[ \frac{\partial \Psi (y,f(\varvec{x}))}{\partial f(\varvec{x})}\right] _{f(\varvec{x})=f_{t-1}(\varvec{x})}. \end{aligned}$$

In this case, the least-squares minimization can be used to replace the potentially very hard optimization task [66]. According to Eqs. 9 and 10, the optimization target for each iteration can be:

$$\begin{aligned} (w_t,\varvec{\theta }_t)=\arg \min \limits _{w,\varvec{\theta }}\sum _{i=1}^{N} [-G_t(\varvec{x}_i) +wh(\varvec{x}_i,\varvec{\theta })]^2 . \end{aligned}$$

#### The process of GBDT

Building GBDT model is an iterative process. Combining the aforementioned CART regression tree and GBM, using the same symbolic representation as before, the main steps are as follows:

Step 1 Initialize the model $f_0(\varvec{x})=\arg \min \limits _{\rho }\sum _{i=1}^{N}\Psi (y_i,\rho )$. In practice, without loss of generality, the mean value of $\{y_i\}_1^N$ can be used instead of $\rho$, that is, $f_0(\varvec{x})={\bar{y}}$.
Step 2 Calculate the gradient of initial loss function at each data, $G_1(\varvec{x}_i)=\left[ \frac{\partial \Psi (y,f_1(\varvec{x}_i))}{\partial f_1(\varvec{x}_i)}\right] _{f_1(\varvec{x}_i)=f_0(\varvec{x}_i)},i=1,2,\ldots ,N$.
Step 3 Use the negative gradient as the label to fit the base learner built in first iteration, $\varvec{\theta _1}=\arg \min \limits _{\varvec{\theta },\rho }\sum _{i=1}^{N}[-G_1(\varvec{x}_i)+\beta h(\varvec{x}_i,\varvec{\theta })]^2$. When the base learner is the CART regression tree model, the fitting result can be recorded as $\{\varvec{R}_{m,1}\}_1^M=M-terminal\,node\,tree(\{\varvec{x}_i,G_1(\varvec{x}_i)\}_1^N)$, that is, which samples are contained in each leaf node of the first tree.
Step 4 With the target of minimizing the loss function of first iteration, find the optimal gradient descent step size, $w_1=\arg \min \limits _w\sum _{i=1}^{N}\Psi (y_i,f_0(\varvec{x}_i)+wh(\varvec{x}_i,\varvec{\theta }_1))$.
Step 5 Update the model $f_1(\varvec{x})=f_0(\varvec{x})+w_1 h(\varvec{x},\varvec{\theta }_1)$. Considering that the base learner is the CART regression tree model, it can be rewritten as $f_1(\varvec{x})=f_0(\varvec{x})+\sum _{m=1}^{M}C_{m,1}\cdot I(\varvec{x}\in \varvec{R}_{m,1})$, where $C_{m,1}=\mathrm{AVE}(-G_1(\varvec{x}_i)\mid \varvec{x}_i\in \varvec{R}_{m,1})$.
Step 6 Repeat Step 2 to Step 5 until the stop condition is satisfied, and then output the final results.

**Fig. 1:**
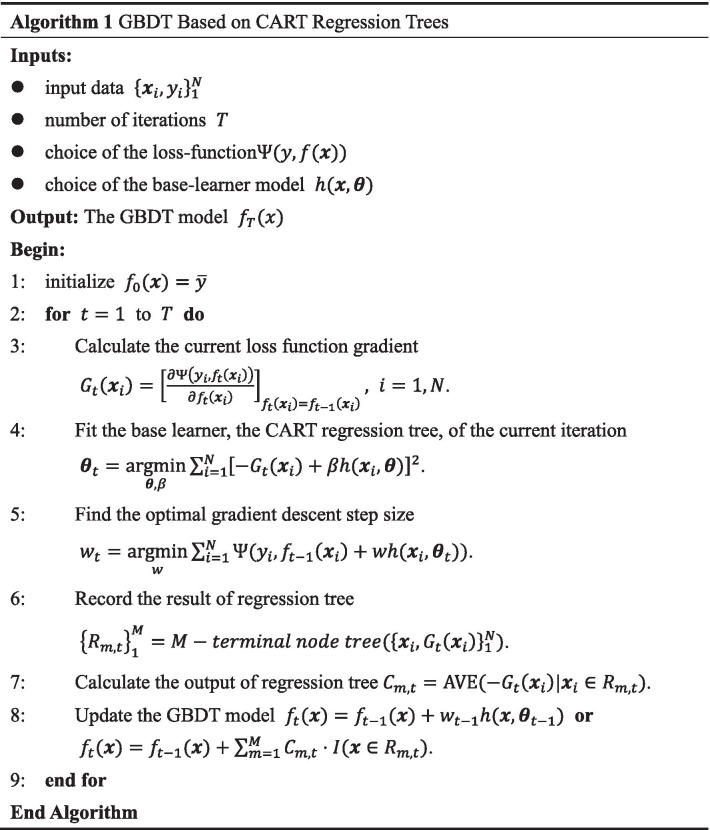
The pseudocode of GBDT algorithm

In Summary, the pseudocode of GBDT algorithm is shown in Fig. 1.

### Feature importance

It should be noted that although “gini value” is not used in partitioning non-leaf nodes in the CART regression tree, we still use the gain of it to evaluate the importance of features, because it is more intuitive and easier to calculate than the change of loss function. Specifically, the importance of a given attribute is evaluated by calculating its reduction in gini values before and after partition of all non-leaf nodes. The more the reduction is, the more important the attribute is. Adding up the importance of this attribute in all trees can obtain its importance in the GBDT model.

In formula form, given a non-leaf node *k*, its gini value $\mathrm{Gini}_k=1-\sum _{i=1}^{I}p_{k,i}^2$, where $p_{k,i}$ represents the proportion of class *i* samples in node *k*, $i=1,2,\ldots ,I$. $\mathrm{Gini}_k$ represents the probability that two samples are randomly selected from *k* with different category labels. Therefore, the smaller $\mathrm{Gini}_k$ is, the higher the purity of *k* is.

Assume that node *k* is split by attribute *c*, and $\mathrm{Gini}_{kl}$ and $\mathrm{Gini}_{kr}$ represent the gini values of the left and right child nodes after splitting, respectively. Then the importance of attribute *c* at node *k* can be obtained as $V_{c,k}=\mathrm{Gini}_k-\mathrm{Gini}_{kl}-\mathrm{Gini}_{kr}$. If node *k* is not split by attribute *c*, $V_{c,k}=0$. Thus, the importance of attribute *c* in the whole tree can be calculated by $V_c=\sum _{k=1}^{K}V_{c,k}$, where *K* is the number of non-leaf nodes in the tree. Then, summing $V_c$ of each tree can obtain overall feature importance of attribute *c* in the whole GBDT model.

### Approach of this paper

In this paper, we presented a new surgical risk calculator based on GBDT named NL-SRC, trying to accurately predict the surgical risk of patients by capturing the non-linear interactions between/among various risk factors. At the same time, in order to accomplish the task well, we also explored the corresponding pipeline for supporting it. In this part, we will introduce these contents from the following main aspects: (1) data preprocessing, (2) missing value filling, (3) parameter adjustment, (4) training mode design, and for simplifying the model by (5) feature dimension reduction. It is worth noting that although these contents are introduced separately in order to make them clear and organized, in practice they are interrelated and interacted with each other, which was be comprehensively considered in our study of the pipeline.

#### Data preprocessing

Generally, the real clinical data collected from hospitals is a mixture of numerical data and categorical data, and there are often repeated records of different data types for the same risk factor. For example, a patient’s white blood cell count (WBC) could have both a numerical record of “$27\times 10^9/L$” and a categorical record of “H” (High), where the former is the quantitative value actually measured, and the latter is the qualitative evaluation made by doctors or experts based on their own knowledge. In addition, there will also be some irrelevant document records (such as admission information and hospital information). Therefore, the clinical data should be preprocessed first.

We first deleted the factors that were extraneous or had the same value in all patients. Then, for the risk factors with duplicate records, we tried three different data combination schemes: all variables, numerical variables mainly and categorical variables mainly. Among them, the best effect was to give priority to categorical variables. Therefore, we retained the qualitative form in duplicate records and deleted redundant data. After that, we coded the factors of category type, mainly following three principles:

1. Each value of a factor should be coded. For example, if factor A had four values $(\mathrm{A}_1,\mathrm{A}_2,\mathrm{A}_3,\mathrm{A}_4)$, they would be coded as (1, 2, 3, 4);
2. For factors whose values represented the degree of severity, coded them in order from low to high. For example, there were three values of urea level: normal (*N*), high (*H*) and very high (*V*), so we coded *N* as 1, *H* as 2 and *V* as 3;
3. For cases where the same value had the same meaning in different factors, coded them in the same coding order, such as all factors that responded with *Yes* and *No*.

#### Missing values filling

For missing values, we tried some methods to fill them, such as mean/mode/median value, interpolation, KNN (K-Nearest Neighbor)-based algorithm and MissForest algorithm, but all with little success. Finally we referred to the missing value treatment method in [68]. The samples that were not missing on the given risk factor were used to find out the split point, then we put the samples with missing into two child nodes and calculated the gains respectively, the direction with larger gain would be selected to split them.

#### Parameters adjustment

The method of parameter adjustment we used was multi-level grid search:

Step 1 Set a value range for each parameter;
Step 2 Selected a small number of parameters and put them into a set. For a given parameter in the set, traversed all the values of it under the condition that other parameters in the set were fixed. Then selected the value with the best result for the given parameter;
Step 3 Repeated Step 2 for each parameter in that set until all parameters obtained their own optimal value;
Step 4 Emptied the set and selected some new parameters to put in, then repeated Step 2 and Step 3, note that the same parameter couldn’t be selected in twice;
Step 5 Repeated Step 2, Step 3 and Step 4 until all parameters were optimized.

#### Training modes design

In order to improve the practicability of our approach, we set up three modes to train the model, making users can flexibly adjust the way to use according to their own data conditions: (1) the cross-validation mode was suitable for the case with a small amount of data; (2) 2:1 mode, that is, the training set and the test set were divided at a ratio of 2:1, which was suitable for general situations; (3) 1:2 mode, that is, the training set and the test set were divided at a ratio of 1:2, which was suitable for situations where the amount of data was large and could be flexibly allocated and combined.

The first two modes are common and easy to understand, and here we will explain the reason for setting training mode 3. In the field of medical risk prediction research, three widely recognized evaluation indicators are generally used to comprehensively evaluate the performance of a surgical risk calculator: area under curve (AUC), Hosmer–Lemeshow test (${\mathrm{HL}}_{\hat{c}}$) and brier score (BS) [13], where AUC is used to measure the discrimination power of the model, and the remaining two are used to measure the degree of calibration. AUC is one of the most common evaluation indicators in various studies, so we will not introduce it in this paper. BS, which examines the overall deviation between the predicted values and the labels from the perspective of the mean, is calculated as $\mathrm{BS}=\frac{1}{N}\sum _{n=1}^{N}(E_n-O_n)^2$, where *N* is the number of samples, $E_n$ and $O_n$ are the predicted value and the label value of the sample *n*, respectively. On this basis, there are two main reasons for continuing to use ${\mathrm{HL}}_{\hat{c}}$ for evaluation: on the one hand, ${\mathrm{HL}}_{\hat{c}}$ can test the significance of this deviation; on the other hand, its sensitivity to the number of samples can help people understand whether the true performance of a method is robust. This is something that the indicators such as AUC and BS, which are relatively stable under different sample sizes, do not possess.

Different from taking the mean, ${\mathrm{HL}}_{\hat{c}}$ examines the calibration of a method by grouping and accumulating the deviations of each group. After obtaining the predicted values in the probabilistic form of the model, all predicted values will be ranked in the order from small to large and divided into 10 groups of equal quantity. Meanwhile, the corresponding label values will also be put into 10 groups. Then, ${\mathrm{HL}}_{\hat{c}}$ can be calculated by the following formula:

11 $$\begin{aligned} {\mathrm{HL}}_{\hat{c}}=\sum _{g=1}^{G}\frac{(O_g-E_g)^2}{N_g\pi _g(1-\pi _g)}, \end{aligned}$$

where *G* is is the number of groups, $O_g$, $E_g$, $N_g$, $\pi _g$ are the sum of label values, the sum of predicted values, the number of samples and the mean of predicted values in group *g*, respectively.

${\mathrm{HL}}_{\hat{c}}$ reflects the degree of deviation of the predicted values from the label values, so the smaller the ${\mathrm{HL}}_{\hat{c}}$, the higher the fitting degree of the two, and the better the performance of the model. From Eq. 11, it is not difficult to find that ${\mathrm{HL}}_{\hat{c}}$ accumulates the deviation between the predicted value of each sample and its label. Since probability prediction methods rarely get results such as 0 and 1, this deviation is common and will accumulate as the sample size increases, thereby resulting in poor ${\mathrm{HL}}_{\hat{c}}$ performance. This characteristic makes ${\mathrm{HL}}_{\hat{c}}$ an indicator that is very sensitive to the number of samples, generally the larger the number of samples included, the worse the performance of ${\mathrm{HL}}_{\hat{c}}$.

Therefore, in conclusion, a surgical risk calculator that can maintain excellent ${\mathrm{HL}}_{\hat{c}}$ performance even with a large sample size can be considered truly accurate and effective. That’s why we set training mode 3 to evaluate the real performance and robustness of our approach.

#### Feature dimension reduction

Considering that a risk prediction system collecting large amounts of physiological data was not only difficult to use in practice [41], but also reduced the willingness of doctors to use it [61], so we calculated the feature importance of each risk factor in NL-SRC and ranked them in terms of importance from high to low. How to calculate the feature importance of risk factors has been explained in the part of *Feature Importance*. Then, we took the top-15 factors to construct new input data and repeated the same training and testing process on it with the same parameters as we did on the original data.

## Results and discussion

The data we used were three-year (2010, 2011, 2012) clinical data from SOMIP [31] launched by the Hospital Authority of Hong Kong, including more than 15,000 cases, 116 risk factors and survival of them within 30 days after surgery. The names and brief information of some risk factors are listed in the tables in the section “Appendix”. We don’t list all of them due to limited space, more detailed information can be found in the official reports of SOMIP [69–71]. Our input data was a matrix of cases and risk factors, where each row represented a case requiring surgery, and each column represented a risk factor, such as age, smoking status and so on. The label reflects to the survival status of each patient within 30 days after the surgery, the death is recorded as 1, otherwise as 0. It is important to note that SOMIP is not a data set for patients with a specific disease but a highly comprehensive surgical data set, which includes all Hospital Authority patients undergoing major/ultra-major procedures in general surgery, urology, plastic surgery and so on [72].

Our experiments were performed in Python3.7 using LightGBM package of Microsoft, and the data we used has been desensitized to delete any data features that might reproduce the patient’s personal information, and does not involve human genetic resource data.

### Results of our approach

We first preprocessed and coded the original data using the steps described before, and finally got 66 risk factors with mixed data types. We studied the results of our approach under the three different training modes preset. Specifically, (1) we used 6-fold and 10-fold cross-validation to perform training mode 1, (2) the data of the previous two years was used as the training set and the rest as the test set to perform training mode 2, (3) the first one year of data was used as the training set and the rest as the test set to perform training mode 3. In addition to our approach, we also built five other models for comparative experiments under the three modes. Among them, the logit model is the most widely used model for surgical risk prediction [32, 41, 57–60], so we regarded it as the most important baseline model to compare and put more attention on its results. Support Vector Machines (SVM) and CART are classic models that are often used in many research fields, and we used them as baseline models to explore the information of baseline values of the evaluation indicators. The remaining two models, Random Forest (RF) and XGBoost, just like the core model GBDT of our approach, are ensemble algorithms based on the tree model, so we use them as representatives of similar models to test whether our approach is superior. With the bold ones being the best under each evaluation indicator respectively, Table 1 shows the results of our approach and three baseline models when using all 66 risk factors, and the results of similar models are shown in Table 2. It is worth noting that we have adjusted the parameters of all the models we used to ensure that they can achieve their best performance.

**Table 1:** Results of baseline models with all risk factors

	Models	AUC	BS	${\mathbf{HL}}_{{{\hat{\varvec{c}}}}}$	P-value
Training mode 1 (10-fold cross validation)	NL-SRC	0.899	0.062	**7**.**398**	**0**.**494**
	logit	0.884	0.065	14.196	0.077
	CART	0.842	0.071	24.850	0.002
	SVM	0.866	0.065	27.490	<0.001
Training mode 1 (6-fold cross validation)	NL-SRC	0.897	0.062	8.798	0.360
	logit	0.883	0.065	13.879	0.085
	CART	0.826	0.070	16.848	0.031
	SVM	0.865	0.064	26.793	<0.001
Training mode 2	NL-SRC	**0**.**902**	0.058	8.391	0.396
	logit	0.890	0.059	12.427	0.133
	CART	0.853	0.065	11.321	0.184
	SVM	0.873	0.059	10.363	0.240
Training mode 3	NL-SRC	0.872	**0**.**047**	15.232	0.055
	logit	0.875	0.066	90.989	<0.001
	CART	0.781	0.077	12.146	0.145
	SVM	0.851	0.068	95.817	<0.001

**Table 2:** Results of similar models with all risk factors

	Models	AUC	BS	${\mathbf{HL}}_{{\hat{\varvec{c}}}}$	P-value
Training mode 1 (10-fold cross validation)	NL-SRC	0.899	0.062	**7**.**398**	**0**.**494**
	logit	0.884	0.065	14.196	0.077
	RF	0.885	0.064	10.426	0.236
	XGBoost	0.895	0.062	13.026	0.111
Training mode 1 (6-fold cross validation)	NL-SRC	0.897	0.062	8.798	0.360
	logit	0.883	0.065	13.879	0.085
	RF	0.885	0.064	12.818	0.118
	XGBoost	0.896	0.063	10.968	0.204
Training mode 2	NL-SRC	**0**.**902**	0.058	8.391	0.396
	logit	0.890	0.059	12.427	0.133
	RF	0.892	0.058	14.226	0.058
	XGBoost	0.900	0.056	9.439	0.306
Training mode 3	NL-SRC	0.872	**0**.**047**	15.232	0.055
	logit	0.875	0.066	90.989	<0.001
	RF	0.879	0.067	11.522	0.174
	XGBoost	0.886	0.067	27.285	<0.001

In evaluating the performance of models, AUC is used to assess the discrimination power of the model and the higher AUC value indicates better performance; BS and ${\mathrm{HL}}_{\hat{c}}$ are employed to measure the calibration of the prediction, or called as the goodness of fit, and smaller values suggest better prediction. The p-value of ${\mathrm{HL}}_{\hat{c}}$ is used to judge whether deviation between predicted values and obvserved ones is significant or not. Therefore, the p-value larger than 0.05 indicates that the model prediction is acceptable and the greater p-value suggests better calibration.

From Table 1, it could be observed that the optimal results (the parts are in bold in Table 1) are all obtained by our approach: the best AUC is obtained under mode 2, which is 0.902; the best BS is obtained under mode 3, which is 0.047; the best ${\mathrm{HL}}_{\hat{c}}$ and the corresponding best P-value are obtained under mode 1 with 10-fold cross-validation, which are 7.398 and 0.494, respectively. In contrast, the best results of the logit model on the four evaluation indicators are 0.890, 0.059, 12.427 and 0.133, respectively. Apparently, our approach is better than the logit model overall. Meanwhile, even if we separately focus on the results in each mode in Table 1, it is not difficult to see that our approach is still better. In both mode 1 and mode 2, NL-SRC outperforms the logit model on all four evaluation indicators, especially in the aspect of calibration degree with significant advantages, which are 7.398 versus 14.196 and 8.798 versus 13.879, respectively. In mode 3, although the AUC of NL-SRC is slightly worse, the performance of ${\mathrm{HL}}_{\hat{c}}$ is far better than the logit model (15.232 vs 90.989). In addition, we can see that as the number of samples included in the test set increases (1530, 2550, 5213, 10280), the ${\mathrm{HL}}_{\hat{c}}$ of NL-SRC and the ${\mathrm{HL}}_{\hat{c}}$ of the logit model rise from 7.398 to 15.232 and from 14.196 to 90.989, respectively. The downward trend confirms the characteristics of ${\mathrm{HL}}_{\hat{c}}$ what we said before and reflects the need of different modes for investigation.

As for SVM and CART, although they are very classic algorithms, they have not been widely used in surgical risk prediction, mainly because their effects in this field are relatively limited, which is consistent with the contents shown in Table 1. Their best AUC is only 0.873, and BS also tends to be higher. Despite in some cases they can show better ${\mathrm{HL}}_{\hat{c}}$ than the logit model (for example, in mode 2), they are still generally inferior and therefore not as good as our approach. However, if we focus on the results of CART, it is easy to find that although its AUCs are not so high, ${\mathrm{HL}}_{\hat{c}}$s almost always maintain a passable performance (10-fold: 24.850, 6-fold: 16.848, mode 2: 11.321, mode 3: 12.146), even better than the traditional logit model in some cases (for example, in mode 2 and mode 3). We think that this implies the potential of tree models in the field of surgical risk prediction, and the key to stimulating this potential lies in how to improve its prediction accuracy through some methods. This is exactly what the original intention of ensemble idea was proposed for, and the results in Table 2 also prove this point.

Table 2 shows the experimental results of the logit model and three ensemble models (our approach, RF and XGBoost). It is easy to see that the four evaluation indicators of the tree models using the ensemble idea are almost better than the logit model in each mode. This not only proves that the non-linear approaches have more advantages in surgical risk prediction, but also illustrates that simplistic models are difficult to solve practical problems in complex systems such as the human physiological environment. The best results of RF (AUC: 0.892, ${\mathrm{HL}}_{\hat{c}}$: 10.426, BS: 0.058, P-value: 0.236) and the best results of XGBoost (AUC: 0.900, ${\mathrm{HL}}_{\hat{c}}$: 9.439, BS: 0.056, P-value: 0.306) are all worse than the best results of our approach (AUC: 0.902, ${\mathrm{HL}}_{\hat{c}}$: 8.391, BS: 0.047, P-value: 0.396). If we pay attention to the results in each mode separately, we can find that our approach is only slightly inferior to RF in mode 3 (AUC: 0.872 vs 0.879, ${\mathrm{HL}}_{\hat{c}}$: 15.232 vs 11.522), and overall is better than the above two models in other cases. This proves that our approach also has some advantages in similar models. We think that the reasons for this situation may be that the performance of RF on regression problems is relatively limited, and the node splitting method of XGBoost limits its ability to capture abundant non-linear interactions.

**Fig. 2:**
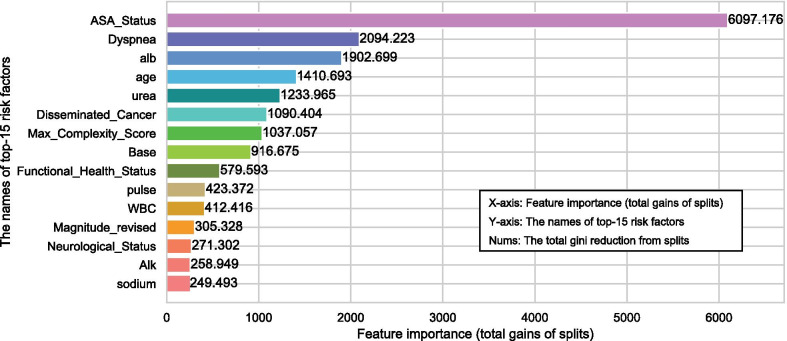
Top-15 most important risk factors and their feature importance

In order to make approaches more practical, we calculated the feature importance of all risk factors and selected the top-15 to construct new data sets for further study. It is worth noting that, except for SVM, we did not adopt a unified top-15 risk factors, but let each model choose important factors for itself. Specifically, the logit model selected factors by the absolute value of the weight of each factor, and CART, RF, XGB and our approach used the feature importance to select. Because it is difficult for SVM to judge the importance of features through the model itself, we used the top-15 risk factors selected by our approach as its input features. Figure 2 shows the top-15 most important risk factors selected by our approach under mode 2. The Y-axis in Fig. 2 represents the names of the selected factors, the X-axis represents the feature importance of them, and the number following each histogram is the specific value of the feature importance of each factor. The larger the value, the more important the factor. The calculation method of these values has been introduced in the part *Feature importance* of *Methods*, that is, one factor’s feature importance equals to the total reduction of gini values caused by the nodes that use the it to implement splitting in the model. Then, similarly, we examined the performance of our approach and the above five models in the three modes on the new data sets. Tables 3 and 4 show the results of baseline models and the results of similar models, respectively.

**Table 3:** Results of baseline models with top-15 risk factors

	Models	AUC	BS	${\mathbf{HL}}_{{\hat{varvec{c}}}}$	P-value
Training mode 1 (10-fold cross validation)	NL-SRC	0.892	0.063	8.082	0.426
	logit	0.864	0.068	13.561	0.094
	CART	0.841	0.069	25.614	0.001
	SVM	0.818	0.069	64.247	<0.001
Training mode 1 (6-fold cross validation)	NL-SRC	0.890	0.064	9.753	0.283
	logit	0.867	0.068	14.603	0.067
	CART	0.825	0.071	20.643	0.008
	SVM	0.820	0.071	55.271	<0.001
Training mode 2	NL-SRC	**0**.**894**	**0**.**060**	**7**.**638**	**0**.**470**
	logit	0.861	0.065	31.460	<0.001
	CART	0.856	0.064	16.088	0.041
	SVM	0.827	0.072	97.202	<0.001
Training mode 3	NL-SRC	0.869	0.066	17.062	0.030
	logit	0.863	0.067	75.033	<0.001
	CART	0.745	0.079	14.266	0.075
	SVM	0.856	0.071	72.822	<0.001

**Table 4:** Results of similar models with top-15 risk factors

	Models	AUC	BS	${\mathbf{HL}}_{{\hat{varvec{c}}}}$	P-value
Training mode 1 (10-fold cross validation)	NL-SRC	0.892	0.063	8.082	0.426
	logit	0.864	0.068	13.561	0.094
	RF	0.881	0.066	12.281	0.139
	XGBoost	0.887	0.064	12.358	0.136
Training mode 1 (6-fold cross validation)	NL-SRC	0.890	0.064	9.753	0.283
	logit	0.867	0.068	14.603	0.067
	RF	0.882	0.066	13.372	0.100
	XGBoost	0.885	0.065	11.726	0.164
Training mode 2	NL-SRC	**0**.**894**	**0**.**060**	**7**.**638**	**0**.**470**
	logit	0.861	0.065	31.460	<0.001
	RF	0.886	0.061	14.022	0.081
	XGBoost	0.882	0.061	9.740	0.284
Training mode 3	NL-SRC	0.869	0.066	17.062	0.030
	logit	0.863	0.067	75.033	<0.001
	RF	0.864	0.069	41.323	<0.001
	XGBoost	0.874	0.069	67.251	<0.001

In Table 3, the global optimal results (the parts are in bold in Table 3) are all obtained by NL-SRC under mode 2, and the AUC, BS, ${\mathrm{HL}}_{\hat{c}}$, and P-values are 0.894, 0.060, 7.638 and 0.470, respectively. Correspondingly, the best results of the logit model are 0.867, 0.065, 13.561, 0.094 respectively, which is obviously not as good as our approach. And in this batch of experiments, the logit model underperforms on the all four evaluation indicators under each mode, and the advantages of NL-SRC in the calibration degree are still very strong. In addition, it can be seen that the increase in the number of samples in the test set still leads to a certain degree of decline in the performance of ${\mathrm{HL}}_{\hat{c}}$, where the ${\mathrm{HL}}_{\hat{c}}$ of NL-SRC and the ${\mathrm{HL}}_{\hat{c}}$ of the logit model rise from 8.082 to 17.062 and from 13.561 to 75.033 respectively. Therefore, it is very necessary to use a large sample volume to test the true effect of a surgical risk calculator. On the other hand, it can be found that the results in Table 3 are generally worse than the results in Table 1, we believe that there should be a strong relationship between the reason for that and the high comprehensiveness of the SOMIP data set. As we mentioned before, this data set contains surgical data from many different surgical fields, and the same risk factor may vary in significance from surgery to surgery. One risk factor will play a key role in some specific types of surgery, in aggregate it may not so important. Therefore, in this data set, each risk factor more or less contributes to the final results, and deleting some of them will inevitably have some negative impact. That’s why the results in Table 3 decline compared to Table 1. Nevertheless, the results are still better than the logit model in all aspects.

As for SVM and CART, the feature dimension reduction also has a significant negative impact on their results. The performance of the four evaluation indicators has consistently declined, especially the ${\mathrm{HL}}_{\hat{c}}$ level of SVM, which even rise to 97.202 at the worst. The conclusions drawn under such performance will be difficult to persuade. In general, their performance is inferior to the logit model and therefore not as good as our approach. In contrast, the results of our approach are more stable, with the same decline but smaller magnitude, and still provide compelling information for doctors and patients. In addition, like the results in Table 1, CART still maintains a relatively stable and passable ${\mathrm{HL}}_{\hat{c}}$ performance (10-fold: 25.614, 6-fold: 20.643, mode 2: 16.088, mode 3: 14.266), and still surpasses the traditional logit model in mode 2 and mode 3 (16.088 vs 31.460, 14.266 vs 75.033). This once again proves the potential of tree models in the research of surgical risk prediction.

Table 4 shows the experimental results of the logit model, RF, XGBoost and our approach under top-15 risk factors. The best performance also comes from the results of our approach under mode 2, which is better than other models in all four evaluation indicators, and still maintains a clear advantage in ${\mathrm{HL}}_{\hat{c}}$ performance, especially in mode 3 which represents a large sample size (17.062 vs 75.033/41.323/67.251). This shows that our approach has higher superiority in similar models. At the same time, even though the input features are reduced by more than 70%, our approach still holds a certain degree of stability, and the magnitude of decline is significantly smaller than the other three models, which proves that our approach not only has a truly excellent ability to predict surgical risk, but also has strong robustness. On the other hand, even when the input features are greatly reduced and the overall effect of each model has declined to a certain extent, from Table 4 we can still draw the conclusion similar to it in Table 2, that is, the results of the three ensemble models are almost still better than the logit model in each mode. This further demonstrates our idea: simplistic models are difficult to solve the practical problems of complex human physiological systems, and the performance of non-linear models will be better than linear models to a certain extent.

### Analysis of selected risk factors

After comparing with the important risk factors listed in the reports of SOMIP [69–71], we found that the factors we selected under mode 2, which are shown in Fig. 2, had a high degree of overlap with them. In our top-15, only two factors, *max complexity score* and *base exceed* level, are important in our research but not in the previous reports.

However, some research have shown that the two factors are closely related to the postoperative survival of patients. *Max complexity score* is the maximum of all the scores given by multiple experts for the complexity of a given surgery. Surgery of different complexity brings different degrees of postoperative risk. For example, although Whipple operation and appendectomy both represent gastrointestinal operations, the postoperative mortality rate of former is significantly higher than latter [73]. Calvete et al. [74] studied severe trauma patients who received surgical treatment in the ICU, and found that *base exceed* level has significant differences between survivors and non-survivors. Therefore, although the risk factors we selected differ somewhat from the reports of SOMIP, there is no doubt that they all play an important role in postoperative risk estimation.

On the other hand, except *magnitude revised*, which is a summary of the severity adjusted for each individual patient, the overlap between the risk factors we selected and those listed in the reports also shows correlation with surgical risk. *Age*, *dyspnea* and *functional health status* are the important causes of bad surgical results in elderly patients with hip fractures [75]; *WBC* is associated with the occurrence of urosepsis which can easily develop into septic shock and lead to death during intra- and post-operative period [76]; *urea* level is related to the mortality of obturator hernia surgery [77]; Alshayeb et al. [78] reported that there is a strong association between the rate of correction of hypernatremia (high blood *sodium* level) and outcome; Gupta et al. [79] believed that *ASA status* is an independent predictor of surgical risk for multiple surgical sub-specialties; the multivariate analysis of [47] determined that hypoproteinemia (low serum *albumin* level) is an independent influencing factor for the risk of emergency surgery in elderly patients; Hu et al. [48] studied 716 patients with gastric cancer and found that lymph node metastasis (the most common way to metastasize of *disseminated cancer*) is associated with postoperative mortality; hypertension (high *pulse* level) is one of the causes of stroke after cardiac surgery [49], which is significantly related to postoperative mortality [80]; Brauer et.al [81] believed that paying attention to neurological complications (*neurological status*) is of great significance for improving the outcome of patients with acute stroke and reducing medical costs; anaplastic lymphoma kinase (*ALK*) is a very important and key driver gene in non-small cell lung cancer [82], and cancers caused by activation of ALK by fusion with other genes are generally highly susceptible to targeted therapy [83], so it has an important impact on the prognosis of treatment.

### Analysis of the interactions between/among risk factors

**Fig. 3:**
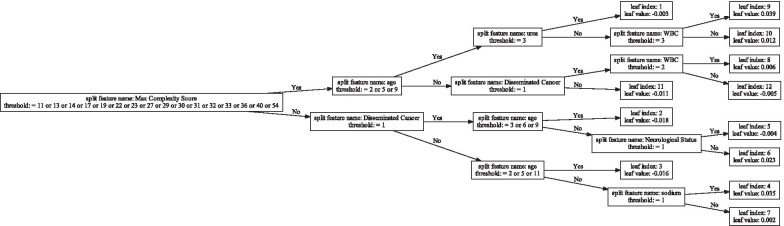
The visualization of the 169th tree with top-15 risk factors under mode 2

In order to examine whether our approach really captured the non-linear interactions between/among multiple risk factors, we randomly chose ten trees in the GBDT model trained with top-15 risk factors in mode 2 and visualized them to analyze their correlations. Here we will take the 169th tree in the GBDT model obtained by our approach under top-15 risk factors as an example for analysis, and Fig. 3 shows the visualization result of it. In Fig. 3, each box represents a node. The boxes that contain the information of the splitting attribute and splitting points represent the root node and the intermediate nodes, and the others who contain the information of their own node serial numbers and node values are the leaf nodes. The node values, marked as “leaf value” in Fig. 3, is the regression results of this tree before sigmoid conversion, so it has both positive and negative values. The directed lines from the left nodes to the right nodes indicate the splitting direction of parent nodes, and the labels next to these lines represent the Boolean relationships between child nodes and splitting conditions, that is, if a sample meets the splitting condition of a parent node, then it will be divided into the child node pointed by the directed line with the label “Yes”, otherwise it will go to the child node pointed by the directed line with the label “No”. For example, if the “Max Complexity Score” of a sample belongs to the set [11, 13, 14, 17, 19, 22, 23, 27, 29, 30, 31, 32, 33, 36, 40, 54], the coded value of “age” belongs to the set [2,5,9], and the coded value of “urea” is equal to 3, then it will be divided into Leaf 1 (that is, the leaf node with “leaf index” 1). This splitting process is actually the decision path at the top of Fig. 3.

It is worth noting that GBDT obtained the final result by adding the results of each tree through the principle of addition, so the result of each tree is incomplete from the overall point, just a fit to its residuals of last iteration. Therefore, although checking the interactions captured in each tree is reasonable, it is not possible to evaluate the effect of the entire model based on the result of a certain tree alone or to use only one tree for prediction.

In Fig. 3, each decision path actually represents some certain non-linear interactions between/among all the splitting attributes involved. Just like the example of Leaf 1, in the process from the root node to Leaf 1, there must be some mutual influences between/among the splitting attributes involved in it (“Max Complexity Score”, “age” and “urea”) around the splitting points. Therefore models can separate eligible samples from other samples by learning them. These mutual influences are actually the interactions between/among risk factors, which will be represented in a non-linear form in GBDT. Most of the interactions in Fig. 3 have some results of relevant research that can be used as supporting evidence. In general, age has a broad and important influence on surgical risk, on the other hand the more complicated the surgery is, the higher the risk is. Kongwibulwut et al. [47] believed that the surgery for elderly patients often has greater surgical complexity, which constitutes a part of the common cause of death after surgery; Hu et al. [48] found both age and the square of age have an important impact on the prognosis of gastric cancer surgery, and reported that there is an interaction between the type of surgery and the size of the tumor, where the latter is profoundly associated to higher lymph node and distant metastasis rate [84]; Bucerius et al. [80] and Arnan et al. [49] respectively reported the impact of age and increased postoperative blood urea nitrogen (BUN) on the risk of stroke after cardiac surgery, we think that preoperative BUN may also have potential influence on it although there is still no conclusion yet; Yoo et al. [85] and Formiga et al. [50] pointed out that WBC and BUN are common laboratory indicators of hyponatremia, which, together with age and hypertension, is an important risk factor for the prognosis of patients with heart failure [85]; Nasr et al. [51] developed the Pediatric Risk Assessment score for non-cardiac surgery with important parameters including but not limited to the age of the patient, the presence of cancers, the status of surgery and the presence of at least one comorbidity (such as neurological or hematological diseases), which has shown high accuracy in the internal validation in a large cohort [86].

The results of the above studies cover most of the interactions in Fig. 3, leaving a few that have not been supported by relevant research. The reason for its appearance may be that our knowledge is limited, or there is some potential connection but not clear yet, or simply because of the incompleteness of a single tree. Nevertheless and on the whole, our approach does capture the non-linear interactions between/among risk factors effectively.

## Conclusions

In this paper, in view of the limitation of existing methods that cannot capture the non-linear interaction between/among risk factors and have encountered the bottleneck of performance, we presented a non-linear surgical risk calculator (NL-SRC) that pioneered the combination of a non-linear ensemble model and surgical risk prediction, and creatively designed an applicable pipeline to give full play to the advantages of GBDT for the characteristics of clinical surgery data. In order to improve the practicality of our approach, we not only set up three different application modes to ensure that users can flexibly adjust the way to apply according to their own data conditions, but also increase the willingness of medical staff to use it by reducing the feature dimensions and model complexity. Experiments conducted on real data demonstrate that our approach has excellent performance. In addition, we also constructed multiple baseline models (logit, SVM, CART) and similar models (RF, XGBoost) and conducted comparative experiments under the same pipeline. The results proves the superiority of our approach. Finally, we analyzed the importance of the selected risk factors and the ability of capturing the non-linear interactions, and the results without exception prove that our approach is effective.

## Data Availability

The datasets are obtained from the surgical outcomes monitoring and improvement project (SOMIP) in Hospital Authorith (HA) of Hong Kong. The datasets generated and analyzed during the current study are not publicly available due to that we have signed a confidentiality agreement with the Hospital Authority of Hong Kong. The data used for research purpose are available from the SOMIP committee in Hospital Authority of Hong Kong upon reasonable request.

## Appendix

### Some information of the dataset SOMIP

Due to limited space, here we only list the names and brief information of some risk factors in SOMIP. The following tables list the statistical information of some numerical data and the number of samples in each category of some categorical data. The names ending in “_num” and ending in “_cat” represent numeric records and categorical records, respectively. More detailed information can be found in the official reports of SOMIP [69–71] (Tables 5, 6).

**Table 5:** Information of some numerical risk factors in SOMIP

Name	Min.	Max.	Median	Mean
Age	1	106	66	62.9
Alb_num	2	56	35	34.41
Alk_num	7	2173	75	93.37
Urea_num	0.1	69.9	5.6	7.479
Base_num	-32	23.9	− 1.1	− 1.788
WBC_num	0.2	91.2	11.13	12.17
Pulse_num	10	985	88	89.21
PCO2_num	0.89	13.99	4.63	4.717
Sodium_num	104	167	137.1	137.1
Max complexity score	0	79	23	23.93

**Table 6:** Information of some categorical risk factors in SOMIP

Name	Categories	Number of samples
ASA Status	1	2704
	2	5828
	3	5405
	4	1278
	5	84
Bloodloss	0	8548
	1	5772
	2	520
	3	194
	4	413
	5	122
WBC_cat	L	256
	N	8220
	H	6570
	M	253
Alb_cat	VL	1910
	L	5618
	N	3964
	H	3419
	M	388
Sepsis	Yes	5323
	No	9976
Disseminated cancer	Yes	1236
	No	14063
Sex	Male	9337
	Female	5962
Current smoker	Smoker	3154
	Ex-smoker	2514
	Non-smoker	9352
Functional health status	Totally dependent	424
	Partially dependent	2033
	Independent	12842
Dyspnea	Dysponea At Re	2077
	Moderate dyspnea	576
	Mild dyspnoea	3248
	No dyspnoea	9398
Magnitude revised	Ultramajor III	1084
	Ultramajor II	1680
	Ultramajor I	2538
	Ultramajor	4
	Major III	2636
	Major II	2995
	Major I	4067
	Major	295

## Abbreviations

NL-SRC: Non-linear surgical risk calculator
GBDT: Gradient Boosting Decision Tree
SOMIP: Surgical Outcome Monitoring and Improvement Program
HSS: The United States Department of Health and Human Services
ASA: The American Society of Anaesthesiologists
APACHE: The Acute Physiology and Chronic Health Evaluation
POSSUM: Physiological and Operative Severity Score for the enUmeration of Mortality and morbidity
P-POSSUM: Portsmouth-POSSUM
BHOM: Biochemistry and Haematology Outcome Model
ACS-NSQIP: The American College of Surgeons National Surgical Quality Improvement Program
SUPPAS: Surgical Risk Preoperative Assessment System
OASIS: The Oxford Acute Severity of Illness Score
HAVM: Healthcare-Associated Ventriculitis and Meningitis
RF: Random Forest
CART: Classfication and Regression Tree
GBM: Gradient Boosting Machine
LSD: Least squares deviation
LAD: Least absolute deviation
WBC: White blood cell count
KNN: K-Nearest Neighbor
AUC: Area under curve
${\mathrm{HL}}_{\hat{c}}$: Hosmer–Lemeshow test
BS: brier score
SVM: Support Vector Machines
ALK: Anaplastic lymphoma kinase
BUN: Blood urea nitrogen
